# Analyzing Various Structural and Temperature Characteristics of Floating Gate Field Effect Transistors Applicable to Fine-Grain Logic-in-Memory Devices

**DOI:** 10.3390/mi15040450

**Published:** 2024-03-27

**Authors:** Sangki Cho, Sueyeon Kim, Myounggon Kang, Seungjae Baik, Jongwook Jeon

**Affiliations:** 1 Department of Electrical and Electronics Engineering, Konkuk University, Seoul 05029, Republic of Korea; kwa3325@konkuk.ac.kr; 2 Department of Electrical and Computer Engineering, Sungkyunkwan University, Suwon 16419, Republic of Korea; sueeyes96@g.skku.edu; 3 Department of Electronics Engineering, Korea National University of Transportation, Chungju 27469, Republic of Korea; mgkang@ut.ac.kr; 4 Semiconductor Research and Development Center, Samsung Electronics, Hwasung-si 18448, Republic of Korea; sea.baik@samgsung.com

**Keywords:** von Neumann bottleneck, logic-in-memory (LiM), floating gate field effect transistor (FGFET), ternary content-addressable memory (TCAM), full adder (FA), temperature, neural network

## Abstract

Although the von Neumann architecture-based computing system has been used for a long time, its limitations in data processing, energy consumption, etc. have led to research on various devices and circuit systems suitable for logic-in-memory (LiM) computing applications. In this paper, we analyze the temperature-dependent device and circuit characteristics of the floating gate field effect transistor (FGFET) source drain barrier (SDB) and FGFET central shallow barrier (CSB) identified in previous papers, and their applicability to LiM applications is specifically confirmed. These FGFETs have the advantage of being much more compatible with existing silicon-based complementary metal oxide semiconductor (CMOS) processes compared to devices using new materials such as ferroelectrics for LiM computing. Utilizing the 32 nm technology node, the leading-edge node where the planar metal oxide semiconductor field effect transistor structure is applied, FGFET devices were analyzed in TCAD, and an environment for analyzing circuits in HSPICE was established. To seamlessly connect FGFET-based devices and circuit analyses, compact models of FGFET-SDB and -CSBs were developed and applied to the design of ternary content-addressable memory (TCAM) and full adder (FA) circuits for LiM. In addition, depression and potential for application of FGFET devices to neural networks were analyzed. The temperature-dependent characteristics of the TCAM and FA circuits with FGFETs were analyzed as an indicator of energy and delay time, and the appropriate number of CSBs should be applied.

## 1. Introduction

The traditional von Neumann architecture has long been utilized in a variety of processors. In the von Neumann architecture, computation is performed through frequent data exchanges between the central processing unit (CPU) and memory. Continuous scaling down has enabled the development of logic complementary metal oxide semiconductor (CMOS) technology, which has improved the speed of CPU operation. However, the speed improvement of memory technology has lagged behind the speed improvement of processing units, resulting in overall system performance degradation. This problem is named von Neumann bottleneck or memory wall [1]. Another important problem is that the processing unit and memory are physically separated, so moving data between them consumes significant energy, causing the overall system to overload. As a way to solve these issues, many studies on in-memory computing have been published recently [2,3,4,5]. Unlike the existing von Neumann architecture, in-memory computing means utilizing technologies such as logic-in-memory (LiM) without moving between memory and CPU. This can reduce unnecessary power consumption and the degradation characteristics of semiconductors compared to traditional architectures. The LiM concept was first proposed in 1970, and the applicability of LiM has been published in the literature using various non-volatile memories such as resistive technologies (ReRAM) [6], magnetic technologies (MRAM) [7], and ferroelectric technologies (FeFET) [8,9]. The LiM concept can be variously divided into technologies such as in-memory computing, coarse-grain LiM, and fine-grain LiM, depending on the location of computation and memory within the system architecture.

The circuits that are applied to the LiM concept include ternary content-addressable memory (TCAM) and full adder (FA) circuits. FA circuits are primarily utilized in digital logic circuits to add two binary numbers. They are used as a key component in the arithmetic logic units (ALUs) of computers, providing high-speed and accurate arithmetic operations for microprocessors, communication equipment, etc. This contributes to improving the performance and efficiency of digital systems. The TCAM circuit is a circuit that can search the stored table and the given data in parallel and check the match on the match line (ML). It has been utilized in applications such as networking hardware, database search, associative memory router, and recently, it has been applied and studied in artificial intelligence architectures [10].

This paper introduces the floating gate field effect transistor (FGFET), which is similar to the floating memory device structure used in existing NAND flash memory, and applies it to LiM applications. In addition, the temperature-dependent characteristics and circuit characteristics of FGFET devices and their potential for use in neural networks were analyzed for the first time. The FGFET structure is based on STTM (scalable two-transistor memory) and PLEDM (phase stat low-electron-number drive random access memory), which were previously announced by Samsung Electronics and Hitachi to replace DRAM and NAND flash memory [11,12,13,14,15,16]. The FGFET stacks a floating node that stores data on the gate of a conventional metal oxide semiconductor field effect transistor (MOSFET), and the write, storage, and read modes of the data are performed by applying voltages to the data line (DL), word line (WL), and sense line (SL). In the storage mode, an energy barrier is placed on the floating node to prevent the data from disappearing. NAND flash is written/erased through a 10 nm thick layer of oxide between the channel and the memory node. FGFETs differ, in that the write/erase mode is driven by current flow according to the VFET’s V_WL_. The retention characteristics of FGFETs are caused by charge leakage in the memory node. The leakage occurs in the tunnelling barrier inserted between the memory node and channel regions of the VFET. If we want to improve the retention time, we can increase the thickness of the corresponding tunneling barrier. However, retention time is a trade-off with write/erase speed. Current AI chips use high-speed memory, such as high bandwidth memory (HBM), around the CPU or graphics processing unit (GPU). We benchmarked the efficiency of logic circuits, etc. with ferroelectric-FETs in references [8,10,17,18] rather than traditional architectures. In this paper, we analyzed the LiM full-adder and TCAM circuits using FGFETs, which are non-volatile (NV) devices. In a conventional AI chip, the GPU is used to compute all the stored vectors, and the HBM stores the vectors. However, TCAM circuits have the advantage of eliminating data movement by calculating the distances in parallel within the memory. NV devices can also be used in full-adder, filp–flop (FF), and static random access memory (SRAM) circuits [9,19,20]. The benefits of using FGFETs in a variety of circuits include increased energy efficiency, real-time processing, reduced data movement, and the reduction or elimination of additional circuits required for volatile circuits. We are confident that this will be beneficial for AI applications.

The device and circuit characterization results of FGFET central shallow barriers (CSBs), which are more advanced than FGFET source drain barriers (SDBs), have been confirmed [21,22,23,24]. Through a device analysis of advanced FGFET-CSBs, it has been analyzed whether structural optimizations can be made in the device itself. Physical temperature changes affect the electrical properties of semiconductor devices, changing the behavioral characteristics of the circuit. These changes affect important metrics such as speed, response time, power, and energy consumption. To see how the change in temperature affects FGFET-SDBs studied at conventional room temperature, the device and circuit characteristics were analyzed at 230 K and 350 K, and the same was done for the FGFET-CSB. Analyzing the temperature-dependent characteristics of each FGFET model is essential for robust and guaranteed circuit characteristics, not only because it allows us to analyze the device characteristics more clearly than in previous papers, but also because it allows us to predict the effects in the circuit. The electrical characteristics of FGFET’s sense-FET (SFET) were verified using well-calibrated TCAD by applying the most scaled-down 32 nm technology node in single-gate planar MOSFET logic process. In addition, FGFET-SDB, FGFET-CSB, and FGFET-2CSB compact models were developed to describe the FGFETs in Synopsys’ HSPICE^TM^. The characteristics of the TCAM and FA circuits with the LiM technology were compared with those of conventional MOSFETs at the existing 32 nm technology node. To verify the applicability of FGFETs to neural networks, the potentiation and depression characteristics of FGFET devices, which represent changes in conductance depending on the number of pulses, were analyzed. We are confident that in this study, we analyzed more diverse factors than previous FGFET papers and confirmed the high potential for industrial application.

This paper is organized as follows: Section 2 is a description of the electrical characterization of FGFET-CSB and SDB models. Section 3 includes temperature-dependent characterization results in TCAM and FA circuits utilizing model libraries for FGFETs. Section 4 is about the possible use of FGFETs in neural networks. Finally, Section 5 provides relevant conclusions.

## 2. FGFET-SDB, -CSB Electrical Properties as a Function of Temperature

**Figure 1:**
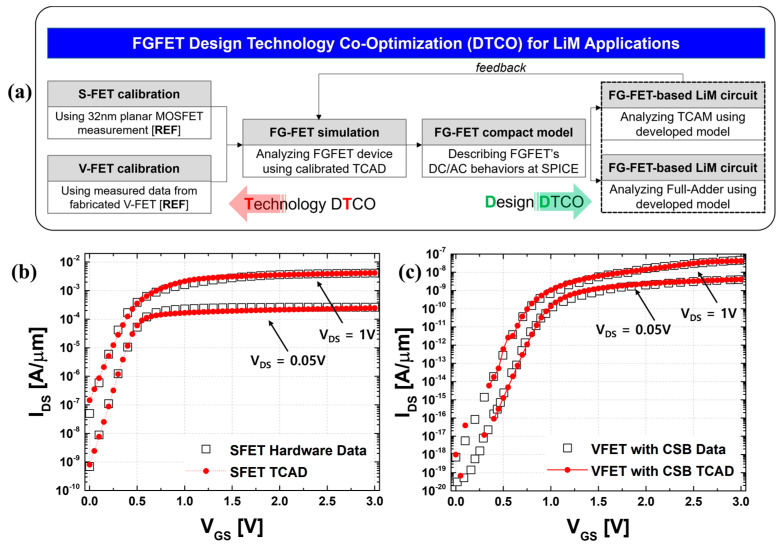
(**a**) Established FGFET design technology co-optimization (DTCO) framework for LiM applications. (**b**) SFET TCAD calibration results with hardware-based I–V transfer curve of planar MOSFET at the 32 nm technology node [25,26]. (**c**) VFET-CSB TCAD calibration results with the hardware-based I–V transfer curve [11].

**Figure 2:**
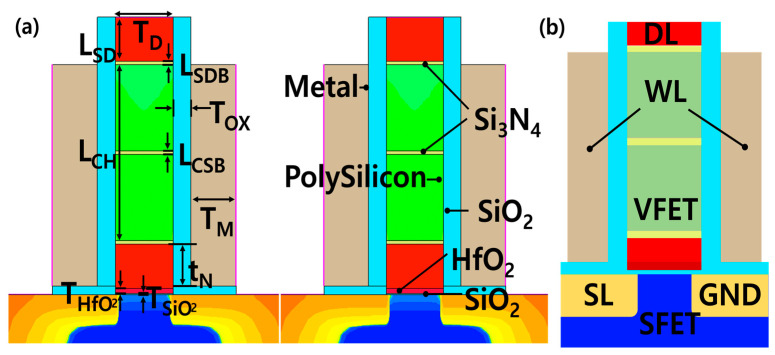
Key device parameters of (**a**) the FGFET with a CSB, (**b**) wiring parameters in FGFET schematic.

In this study, we analyzed the use of Synopsys Sentaurus^TM^ TCAD [25]. Effective calibration was achieved by stacking a vertical-FET (VFET) on the gate of the SFET in the same way as in a previous paper [23]. The SFET has a similar structure to a conventional planar MOSFET and is fabricated in 32 nm technology, which is the most advanced process for planar MOSFETs. The SFETs were TCAD calibrated using the predictive technology model (PTM) developed by Arizona State University (ASU) [26,27,28,29,30,31]. The SDB and CSB devices of VFET are most affected by tunneling due to the presence of both barriers. Therefore, the calibration is centered on the tunneling mass density of state (DOS), and the subthreshold swing is fitted through work function engineering and interface trap adjustments. Afterward, minority elements are fitted with the drift-diffusion current model, and the above method is repeated to improve the device accuracy. Figure 1 shows the overall flow chart of proper FGFET calibration and design technology co-optimization (DTCO) for the circuit with FGFETs described later. Figure 2 shows the structure and material of the FGFET, and Table 1 shows the key parameters of the FGFET model. To analyze the retention time (RT) and memory window (MW) of the FGFET-CSB, -SDB model, we utilized the values of t_N_ and L_CH_ presented as optimal parameters in previous papers and in Table 2 [23].

### 2.1. The Retention Time of FGFET-CSB and SDB

**Figure 3:**
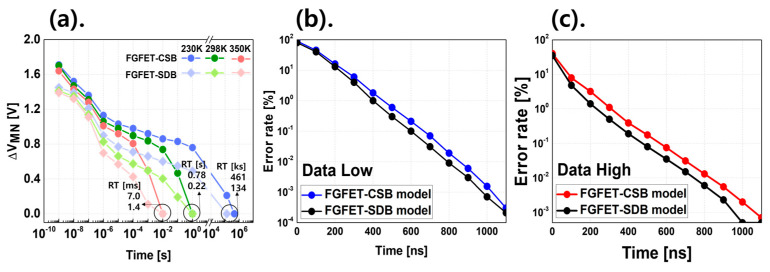
(**a**) RT characteristics as a function of temperature for the FGFET-SDB model and CSB model and write speed by the FGFET model converted to error rate. (**b**) Data low and (**c**) data high.

**Figure 4:**
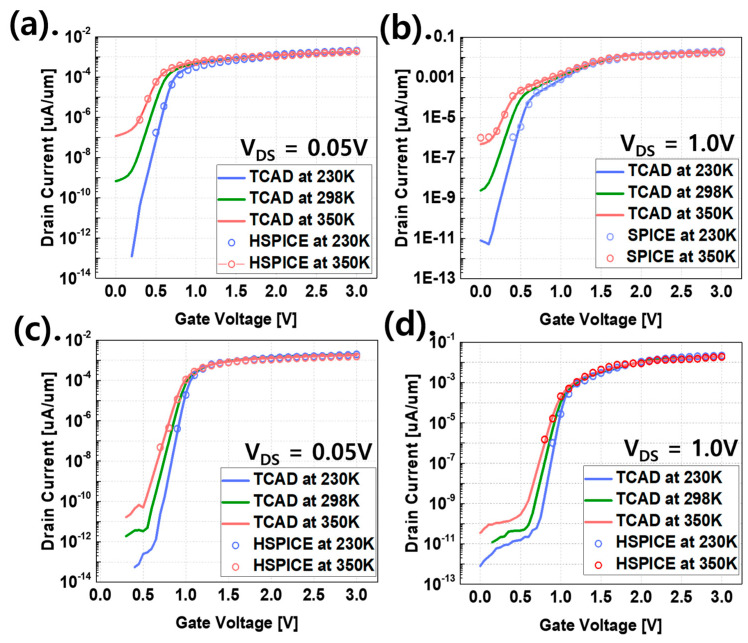
FGFET-SDB model I_D_–V_G_ curve. (**a**) V_DS_ = 0.05 V, (**b**) V_DS_ = 1.0 V, and FGFET-CSB model I_D_–V_G_ (**c**) V_DS_ = 0.05 V, and (**d**) V_DS_ = 1.0 V.

RT indicates how long the data are stored without applying voltage to all the nodes while writing data ‘0’ or ‘1’ to the memory device. To measure the RT of the FGFET, we write each data to the FGFET and calculate the voltage difference across the memory nodes (ΔV_MN_), which is the voltage difference between data ‘0’ and ‘1’ in the turn-off state [23]. Figure 3 shows the RT of the FGFET-SDB and FGFET-CSB models as a function of temperature, as well as the write speed at room temperature. From Figure 3, it can be seen that retention time and write/erase speed are in a trade-off relationship. The FGFET-CSB model has a 244% improvement over the FGFET-SDB model at a temperature of 230 K, a 255% improvement at room temperature, and a 400% improvement at the high temperature of 350 K. The FGFET-CSB model has a better RT than the FGFET-SDB model in all cases. The reason for the better RT characteristics of the FGFET-CSB is explained in Figure 4. RT is the state in which no voltage is applied to WL, DL, and SL and is very relevant to VFETs. Figure 4 shows the I–V curves of the VFETs that are part of the FGFET and shows that the VFET-CSB model has a lower off-current at all temperatures than the VFET-SDB model. The leakage current is controlled by restricting the movement of electrons due to the CSB, and the off-current of the FGFET-CSB and FGFET-SDB at 350 K is the most different by an order of four, which is a high improvement rate. However, at 230 K, the off-current is very small by an order of one, which is the lowest improvement rate.

### 2.2. The Memory Window of FGFET-CSB and SDB

MW is a concept for evaluating the stability and reliability in semiconductor memory technology, indicating the extent to which a memory cell will operate reliably and store data correctly under certain conditions. It indicates the robustness to changes in the external environment, and its size is an important metric for evaluating the reliability of a memory device. MW allows for the evaluation of the performance of a device and the determination of the extent to which it can respond to different environmental conditions.

The MW of each model was calculated using Formula (1), since the voltage to store data ‘1’ is less than the voltage to read data ‘0’. The FGFET-CSB has a better MW than the conventional model at all temperatures. This is because the central barrier of the VFET-CSB does not affect the movement of electrons in the on-current case but does prevents them from escaping in the off-current case. Therefore, the performance of the FGFET-CSB with the advanced VFET-CSB is better than the FGEFT-SDB with the conventional VFET. Table 3. shows the MW as a function of temperature for the FGFET model. The MW of the FGFET-SDB model is smaller than that of the FGFET-CSB model at all temperatures.

V_H_(S) < V_L_(R) → MW = V_H_(R) − V_L_(R), (1)

## 3. FGFET Compact Modeling and Circuit Characterization for LiM Applicability

**Figure 5:**
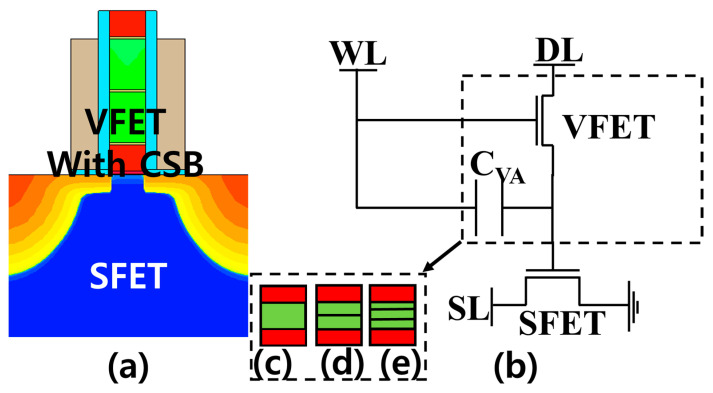
FGFET with CSB (**a**) cross section view and (**b**) equivalent circuit model, integrating a VFET on the gate of SFET. (**c**) No CSB-VFET, (**d**) one CSB-VFET, and (**e**) two CSBs-VFET.

Temperature-specific compact models of various FGFET devices and FGFET-based LiM circuits, TCAM, and FA circuits were analyzed. As for the FGFET devices, FGFET-SDB, -CSB, and FGFET-2CSBs with two CSBs in the channel were also evaluated using the FGFET-CSB implemented in TCAD. This also confirmed the impact of the number of channel barriers on the FGFET. FGFETs consist of a VFET, an SFET, and a coupling capacitor (C_VA_) that is generated in the process. The VFETs were created based on the BSIM4 model, and the SFETs were modeled using ASU’s PTM and were modeled according to temperature by adjusting the parameters of the PTM [30,31]. The C_VA_ was modeled using Verilog-A to consider not only the physical capacitance due to the dielectric layer but also the depletion capacitance considering the voltage condition to improve the matching with each mode characteristic of the FGFET. Figure 5 shows the cross section and equivalent circuit of the FGFET.

**Figure 6:**
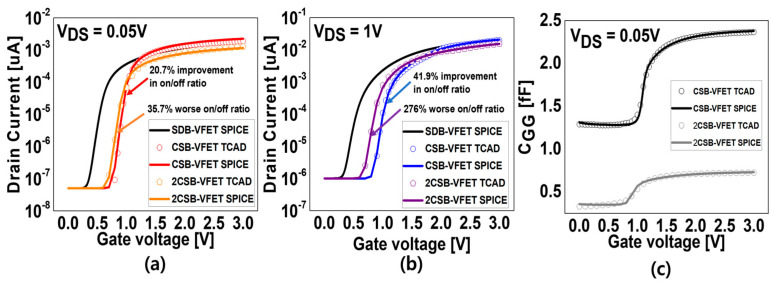
Comparison of the simulation CSBs-VFET’s results between the TCAD and SPICE model for (**a**) I_DS_–V_GS_ at low V_DS_, (**b**) I_DS_–V_GS_ at high V_DS_, and (**c**) C_GG_–V_GS_ at low V_DS_.

Figure 6 shows the I_DS_–V_GS_ curve and C_GG_–V_GS_ curve for the CSB and 2CSBs with the same off-current as the conventional VFET. For a V_DS_ voltage of 0.05 V, the on/off ratio in I_DS_–V_GS_ is improved by 20.7% for the CSBs-VFET compared to the SDB-VFET, but it is degraded by 35.7% for the 2CSBs-VFET. Also, at a V_DS_ voltage of 1 V, the same as at 0.05 V, the CSB-VFET improved by 41.9%, while the 2CSBs-VFET deteriorated by 276%. The V_WL_ modulates the internal potential of the intrinsic silicon region, and the CSBs move up and down energetically with the internal potential. The 2CSBs have a higher energy band level than the CSBs, which prevents electrons from moving from the source to the drain of the VFET, resulting in a decrease in the linear region (on-current) [15]. The temperature-dependent VFET models are also modeled as shown in Figure 6, which is confirmed in Figure 4. For C_VA_, the capacitance of the geometric direct overlap area, the depletion region capacitance, and the capacitance due to the fringe field are considered.

**Figure 7:**
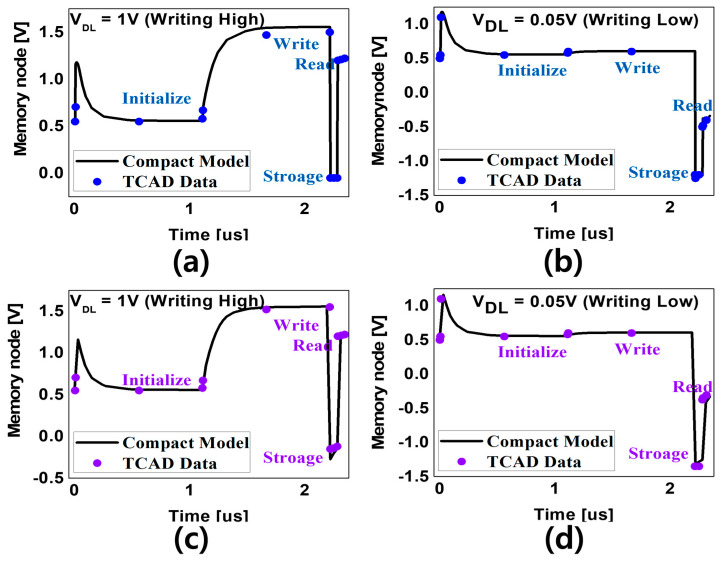
FGFET compact model: one CSB-FGFET (**a**) one-bit memory timing chart at V_DL_ = 1 V, (**b**) one-bit memory timing chart at V_DL_ = 0.05 V. Two CSBs-FGFET (**c**) one-bit memory operating chart at V_DL_ = 1 V and (**d**) one-bit memory operating chart at V_DL_ = 0.05 V.

Figure 7 shows the transient characteristics of a compact model incorporating VFETs, SFETs, and C_VA_, considering the above points. This shows the agreement of the compact model with the TCAD results and its characteristics under each operating condition. Figure 7a,b show the FGFET-CSB model, which has the same operating voltage conditions as the FGFET-SDB model [21]. However, the FGFET-2CSBs model with the results shown in Figure 7c,d has a problem. If −2 V is applied to the VWL in storage mode, the VMN becomes larger than 0 when the VDL is high and data are not stored. To solve this problem, the model has been adjusted to apply −2.5 V to the VWL in storage mode. Also, in this study, we cannot guarantee sufficient RT in LiM computing in the same way as in Section 2.1. As shown in Figure 7, after writing, we applied a separate behavior by applying −2 V to the V_WL_ in the storage mode. This ensures that the FGFET has enough retention time.

### 3.1. TCAM, FA Circuit Characteristics with and without Central Shallow Barriers

**Figure 8:**
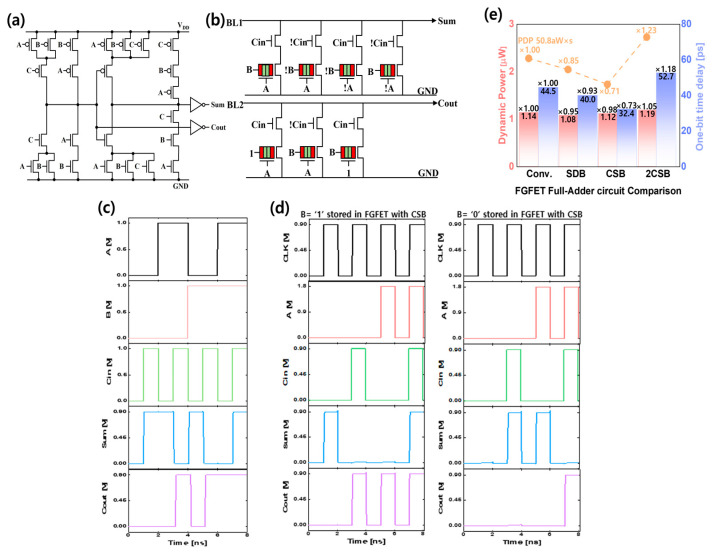
(**a**) Conventional 28 FET, (**b**) 13 FET + 7 FGFET full adder (FA) circuit. (**c**) Timing graph of conventional 28 FET FA, and (**d**) timing graph of 13 FET + 7 FGFETs FA (B = 1 and 0 stored in FGFET). (**e**) Performance and power of FA.

In this section, TCAM and FA circuits with FGFETs were analyzed. When non-volatile elements are applied in FA, fast data access and low-power high-performance circuits can be realized [32,33]. Figure 8 shows a conventional 28 transistor (28 FET)-based FA as the SFETs used in the baseline of the FGFET and a 13 FET + 7 FGFETs FA implemented with 13 transistors and 7 FGFETs [34]. This includes a schematic, timing diagram, and performance analysis. For the 13 FET + 7 FGFET, V_WL_ and V_DL_ are used as input terminals A and B, respectively. V_WL_ is applied at 1.8 V and 0 V complementarily in the read mode, and V_DL_ is applied at 1.8 V for data ‘1’ and 0.1 V for data ‘0’. After writing and storing the desired data to the FGFET, the FA operates in read mode with the data ‘0’ or ‘1’ stored at input terminal B. The threshold voltage (V_th_) of the VFET-CSBs was shifted by −0.4 V, and the V_th_ of the VFET-2CSBs was shifted by −0.3 V to move the I_DS_–V_GS_ curve of the VFETs to the negative region so that the FGFETs can perform the AND operation. The V_th_ of the SFET was shifted by 0.8 V. The V_DD_ of the 28 FET FA is set to 0.9 V. In the LiM architecture, FA performs the operation by reading the stored data. Therefore, the performance of the read mode is important, so we evaluated the performance of this mode. The performance evaluation included measurements of the delay time (T_D_), dynamic power (P_DYN_), and power delay time product (PDP). Comparing the FGFET-based FA to the 28 FET FA, the T_D_, P_DYN_, and PDP of the FGFET-CSB-based FA are improved by 2%, 27%, and 29%, respectively. However, the FA based on FGFET-2CSBs degraded T_D_, P_DYN_, and PDP by 5%, 18%, and 23%, respectively. Unlike the FGFET-2CSBs-based FA, the FGFET-CSB-based FA consumes more dynamic power in the read mode than the FGFET-SDB-based FA due to the larger current variation with V_MN_. The delay time of the FGFET-CSB-based FA improves over the FGFET-SDB-based FA as the power consumption increases.

**Figure 9:**
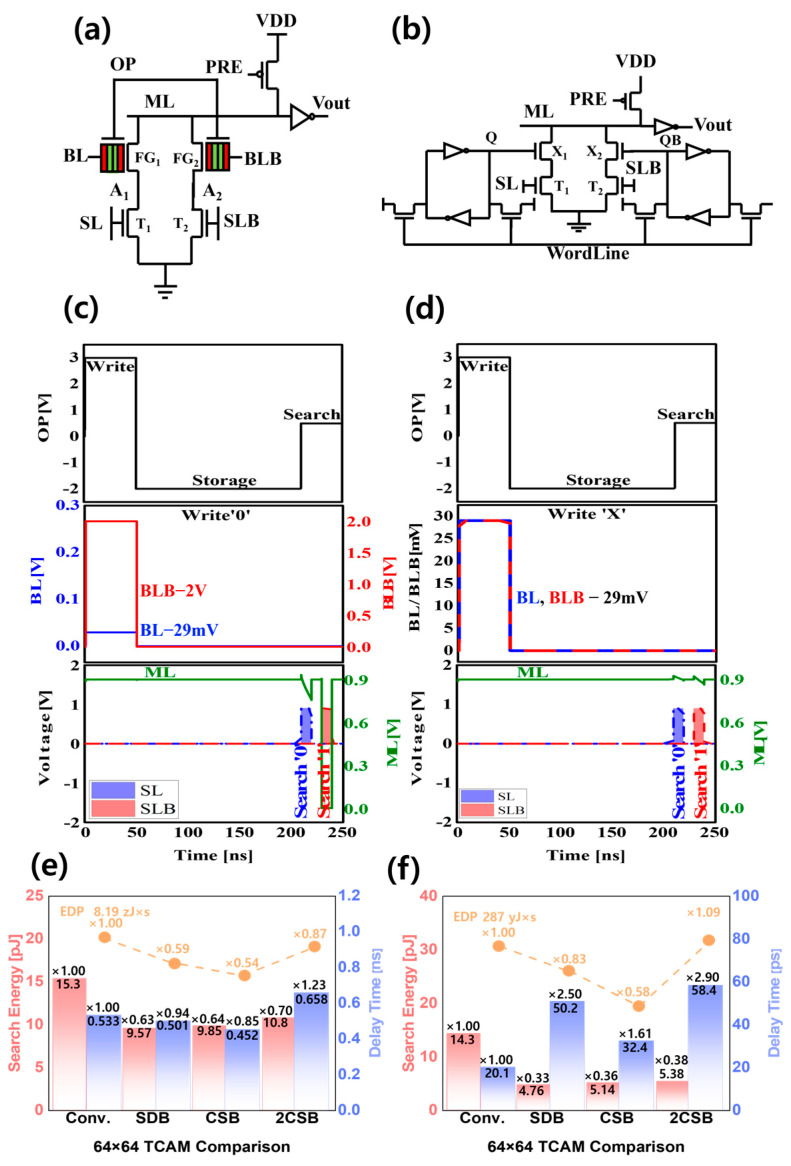
(**a**) 2 FET + 2 FGFET TCAM, (**b**) conventional 16 FET TCAM schematic. (**c**) In 2 FET + 2 FGFET TCAM, when write is 0, search is 0 and 1; (**d**) when write is x, search is 0 and 1. (**e**) In the case of ‘1-mismatch’, the TCAM characteristic comparison results, and (**f**) in the case of ‘all-mismatch’, the TCAM characteristic comparison results.

The TCAM circuit is a high-speed application for finding data stored in FGFETs. It searches the data ‘0’, ‘1’, and ‘don’t care’ states in parallel and presents the results. Recently, TCAM circuits have been applied to neural networks and computing [35,36,37]. Figure 9 shows the schematic, timing diagram, and performance evaluation of a TCAM circuit using 2 transistors and 2 FGFET (2 FET + 2 FGFET) and a TCAM circuit with 28 conventional transistors (28 FET), including delay time, etc. in the search mode. In the previous paper [21], the time of the storage mode was set to 50 ns, but in this study, the time of the storage mode was changed to 160 ns to clearly see the performance difference with temperature of the FGFET model, which will be discussed in Section 3.2. To evaluate the FGFET-SDB model at the same V_th_ as the TCAM of the FGFET-SDB model, the V_th_ of the VFET-CSB and VFET-2CSBs models was adjusted to be the same as that of the FA circuit implementation, and the V_th_ of the SFET was shifted by 0.2 V. At a high V_DS_ voltage, the VFETs in the shifted FGFET-CSB model showed a 26.4% improvement in the current change at the same voltage over the conventional VFETs, while the VFETs in the FGFET-2CSBs model showed a 16.3% deterioration. At low V_DS_ voltages, the VFET-CSBs improved by 18.2% and the VFET-2CSBs deteriorated by 29.9% compared to conventional VFETs. Having different current levels for different VFETs causes the ΔV_MN_ of the FGFETs, which affects the delay time, search energy (E_S_), and energy delay time product (EDP), which are the performance evaluation factors of TCAM circuits. Therefore, in 1-match, Figure 9e, the E_S_, delay time, and EDP of the FGFET-CSB TCAM are improved by 15.2%, 35.6%, and 45.4%, respectively, compared to the 28 FETs TCAM in 1-mismatch. On the other hand, the FGFET-2CSB TCAM improved E_S_ and EDP by 29.4% and 12.9%, respectively, compared to the 28 FET TCAM, but the delay time was 23.4% worse. Compared to the FGFET-SDB TCAM, the FGFET-2CSBs TCAM was worse across all the metrics. Figure 9f shows the overall performance in all-mismatch. As with the 1-mismatch case, the FGFET-2CSBs-based TCAM performed worse than the FGFET-SDB-based TCAM. Compared to the 28 FET-based TCAM, the FGFET-2CSBs TCAM improved E_S_ by 62.4%, but the delay time and EDP degraded by 191% and 9.31%, respectively. The FGFET-CSBs-based TCAM had a 38.8% worse delay time, but 64.1% better E_S_ and 42.1% better EDP.

### 3.2. TCAM, FA Circuit Characteristics of FGFET Devices by Temperature

**Figure 10:**
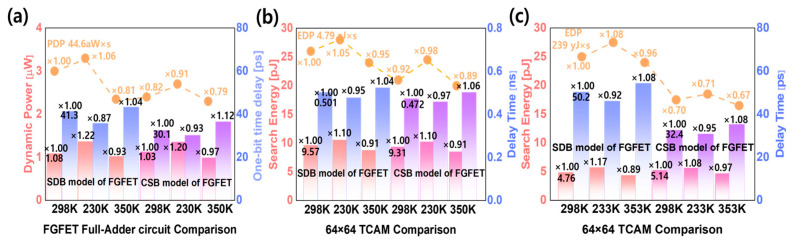
Performance of FGFET-SDB, -CSB models as a function of temperature. (**a**) FA circuit, (**b**) under ‘1-mismatch’ and (**c**) TCAM circuit under ‘all-mismatch’.

Physical temperature changes affect the electrical properties of semiconductor devices, which are related to voltage, current, conductivity, and electrical resistance. Therefore, temperature changes directly affect the behavioral characteristics of a circuit, which can lead to changes in important metrics such as speed, response time, and power consumption. Figure 10 shows the circuit characteristics at 230 K, 298 K, and 350 K for the FGFET models. The TCAM and FA circuits improved at lower temperatures, while P_DYN_ and E_S_ increased at higher temperatures. In Figure 4, the ion in the VFET is the largest at 230 K. Therefore, the delay time is proportional to the temperature, and P_DYN_ and E_S_ are inversely proportional to the temperature.

## 4. Neural Network Availability for FGFET Devices

**Figure 11:**
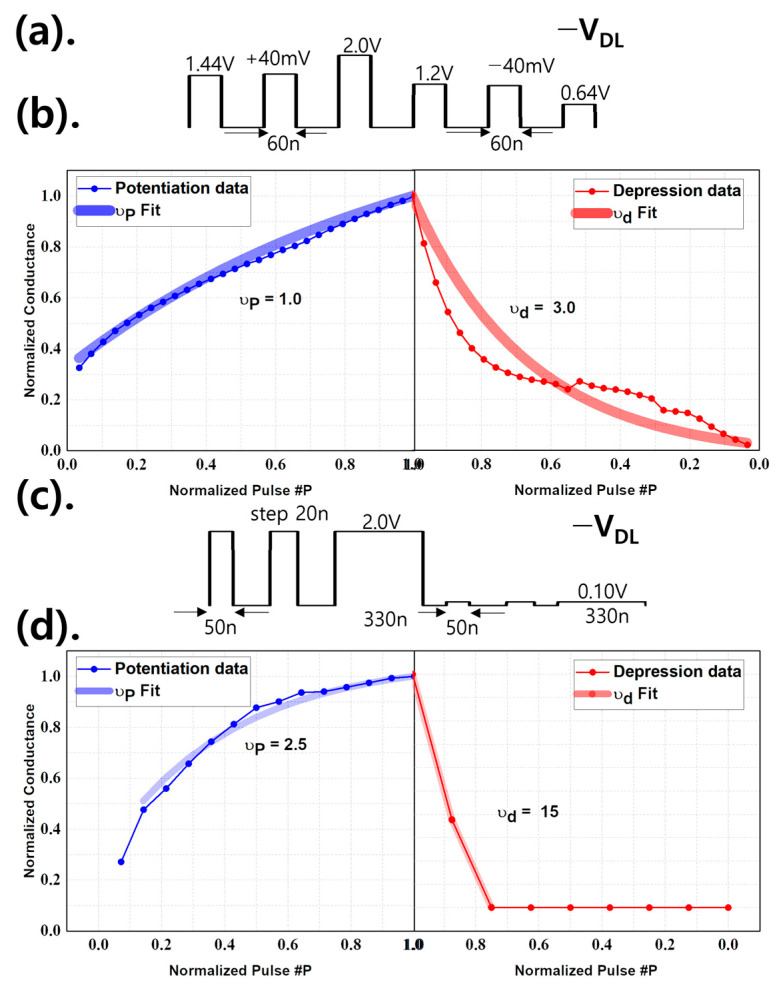
Voltage application method of the V_DL_ of the FGFET-SDB model and the value of the normalized conductance according to the pulse. (**a**) Scheme 1 for the method using V_DL_ voltage difference. (**b**) Potentiation and depression according to scheme 1. (**c**) Scheme 2 using pulse width difference. (**d**) Potentiation and depression according to scheme 2.

Neuromorphic platforms have garnered considerable attention as a new computing system that goes beyond traditional von Neumann architectures due to their high efficiency, low power consumption, and adaptive and parallel signal processing [38]. Since the neural network is bio-inspired, the device requires long-term depression (LTD) and potentiation (LTP), two synaptic functions that are essential for learning [39,40,41]. In this section, the potential of FGFETs in neural networks has been analyzed. Figure 11 shows the quantitative analysis of the linearity of the weight update behavior in the LTP/LTD process of the FGFET-SDB model. The nonlinearity factor (ν), which represents the nonlinear behavior of the weight update, is calculated based on the normalized conductance (G_P_ or G_D_) as a function of the number of pulses (p) [42]:

(2) $$G_{P}=G_{min}+B\times (1-e^{-\nu \times p})$$

(3) $$G_{D}=G_{max}-B\times (1-e^{-\nu \times (p-p_{max})})$$

(4) $$B=\frac{G_{max}-G_{min}}{1-e^{-\nu \times P_{max}}}$$

**Figure 12:**
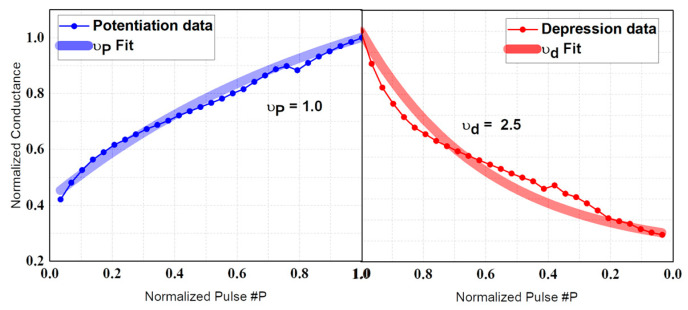
Conductance as a function of number of pulses for FGFET-CSB model using the Scheme 1 method.

Figure 11a shows the cases where the V_DL_’s pulse width is kept the same, and the voltage is increased by 40 mV in potentiation and decreased by 40 mV in depression to retrieve the conductance value. Figure 11b shows the conductance by applying a voltage using the method in Figure 11a, and the conductance is plotted by applying a voltage. Figure 11c shows the case where the V_DL_ value is kept the same and the period of the pulse is increased by 20 ns, from which Figure 11d was extracted. The smaller the absolute value of ν is, the better the linearity. Potentiation is no different, but depression is where scheme 1 is superior. This is because FGFET devices are closely related to the tunneling phenomenon and are therefore more affected by voltage-dependent barriers than pulse width. Figure 12 shows the potentiation and depression of the FGFET-CSB model. Comparing Figure 11 and Figure 12, the FGFET-CSB model has good linearity and is therefore suitable for a neural network.

## 5. Conclusions

In this paper, various FGFET models were characterized. Compared to the FGFET-SDB model, the FGFET-CSB model was superior in terms of RT and MW at all temperatures. The TCAM and FA circuits using HSPICE consumed more power/energy because the VFETs in the FGFET-CSB model have higher on-current than the VFETs in the FGFET-SDB model. However, FGFET-CSB was superior in PDP and EDP, which comprehensively evaluate power/energy and performance, due to the improvement of FGFET-CSB in terms of performance. However, the FGFET-2CSB model exhibited worse characteristics than the FGFET-SDB and FGFET-CSB models in all aspects. In this respect, a reasonable number of CSBs is required to properly evaluate the FGFET model.

## Figures and Tables

**Table 1:** Values for key device parameters of the FGFET with CSBs in this work.

Parameters	Values
Gate separation (T_D_)	32 nm
VFET gate oxide thickness (T_ox_)	10 nm
Metal thickness (T_M_)	25 nm
VFET channel length (L_CH_)	100.2 nm
Source/drain length (L_SD_)	25 nm
Source/drain barrier (L_SDB_)	2 nm
Central shallow barrier (L_CSB_)	2 nm
VFET channel doping	Intrinsic
VFET S/D doping	2 × 10^20^ cm^−3^
Memory node thickness (t_N_)	23.7 nm
SiO_2_ thickness (TSiO_2_)	0.7 nm
HfO_2_ thickness (THfO_2_)	3 nm
Substrate doping	1.0 × 10^16^~1.8 × 10^16^ cm^−3^
SFET source/drain doping	5 × 10^19^ cm^−3^

**Table 2:** Voltage conditions for FGFET model operation modes.

Mode	V_WL_ [V]	V_DL_ [V]	V_SL_ [V]
Initialize	3	0	0
Write	3	0.05 (low, Data ‘0’)/1 (high, Data ‘1’)	0
Storage	−2	0	0
Read	0.5	0	0.9

**Table 3:** Memory windows for FGFET models as a function of temperature.

	SDB Model			CSB Model		
TEM	230 K	298 K	350 K	230 K	298 K	350 K
MW [V]	1.16	1.14	1.12	1.24	1.22	1.21
Increase/decrease Rate [%]	1.75	0	−1.75	8.77	7.02	6.14

## Data Availability

Data is contained within the article.
